# Executive Functions in Tobacco Use Disorder: New Challenges and Opportunities

**DOI:** 10.3389/fpsyt.2021.586520

**Published:** 2021-03-15

**Authors:** Raquel Martín Ríos, Francisca López-Torrecillas, Ignacio Martín Tamayo

**Affiliations:** ^1^Department of Personality, Assessment and Psychological Treatment, University of Granada, Granada, Spain; ^2^Department of Methodology of Behavioral Sciences, University of Granada, Granada, Spain

**Keywords:** smoking, neuropsychologial assessment, executive function, working memory, tobacco

## Abstract

There is increasing evidence that executive functions have significative effects on nicotine abuse. An unresolved challenge for smoking cessation interventions is the detection of factors associated with nicotine use. In order to understand how cognition is affected by nicotine abuse, this study was designed to determine the relationship between years of smoking addiction and several variables of executive functions. The sample was composed of 174 smokers, whose age ranged between 27 and 69 years old (M = 47.44; SD = 8.48). Smokers were assessed at baseline with measures of cognitive inhibition [Go/No Go Task and Five Digit Test (FDT)], updating [Visual Search and Attention Test (VSAT) and Letter-Number Sequencing (WAIS IV)] and shifting [Delay Discounting Task (DDT) and Iowa Gambling Task (IGT)] while the outcome measure was years of smoking. The linear regression and correlation analysis highlighting that the variable which has the strongest association with years of smoking is updating. Multivariate analysis of variance (MANCOVA) followed by Tukey *post-hoc* tests revealed significant differences such that heavy smoking indicated worse performance than light smoking on updating tasks. These findings report the ability of working memory to predict years of smoking and suggest that cigarette packaging warning may experience a loss of effectiveness in heavy smokers.

## Introduction

Tobacco use is a major risk factor for cardiovascular and respiratory diseases (1). Smoking is also unfavorable to the immune systems, making smokers more vulnerable to infectious diseases (2). Despite this, the tobacco industry seems unperturbed (3). An unresolved challenge for smoking cessation interventions is the detection of factors associated with nicotine use. In particular, findings in animal models suggest that the underlying reasons for smoking may be quite different across individuals (4).

Several factors are identified to have an impact on smoking addiction: age of initiation (5, 6), negative affective states (7–9), mood disorders (10), psychiatric disorders (11, 12) and executive functioning (8, 13–15). The addiction process elicits neuroadaptations that affect broadly distributed neural circuits involved in cognitive process (16). Indeed, there is increasing evidence that executive functions have significant effects on nicotine dependence. Executive function (EF) refers to a “*set of general-purpose control processes that regulate one's thoughts and behaviors”* (17). Miyake et al. (18) identified three different components of EF: (1) Inhibitory processes, (2) Updating, and (3) Shifting processes (18). However, studies examining associations between executive measures and clinical aspects of smoking such as years of smoking addiction are reduced and generally limited to predict risk profiles. Also, existing literature examining the effects of EF on substance abuse focuses on inhibition and working memory components (14).

Flaudias et al. (13) evaluated the influence of executive functions (inhibition, updating and shifting) on craving [The Tobacco Craving Questionnaire; (19)] and nicotine dependence [Fagerström Test for Nicotine Dependence, (20)] in a sample of moderate (Fagerström >3) and severe (Fagerström >7) smokers. They used a Stroop task (21) and Hayling's test (22) as measures of inhibition, “Trail Making Test” (23) as a measure of shifting, and the “N-back” test (24) for updating. The dimension of inhibition seems to be a significantly stronger predictor of tobacco dependence than craving or updating abilities (13). Findings are reminiscent of the work of Billieux et al. (25) who has discovered that a poor ability to inhibit evaluated by a go-stop task (26) as well as age are significant predictors of tobacco dependence evaluated by Fagerström Test for Nicotine Dependence, (20). Further, Hu et al. (27) examined the relationship between smoking and cognitive performance in working memory among smokers and non-smokers. All participants were evaluated with the subtests of information, arithmetic and digits (28) and the Dysexecutive Questionnaire (29). In addition, smokers were measured on their level of nicotine dependence (20). Smokers reported lower performance on the arithmetic and digit subtests than non-smokers. There was also a direct correlation between digit scores (reverse order condition) and age of smoking initiation. On the other hand, smokers had higher scores on the dysexecutive questionnaire than non-smokers (27). Taken together, poor executive functions have been linked to nicotine abuse but it is unclear whether such executive components differentially interfere with each stage of addiction. Furthermore, to our knowledge, there are no studies that explore years of smoking addiction as a possible factor linked to executive performance.

Studying executive measures together with behavioral aspects of smoking could be of major interest for several reasons. Firstly, smoking cessation therapies are ineffective for most treatment-seeking smokers (30). A better understanding of the underpinnings of nicotine use may help to improve smoking cessation treatment. Secondly, several studies advocate the idea of including further smoking history variables because they would provide possible illustrative predictors of correlates of nicotine dependence (31, 32). Finally, exploring neuropsychological measures among different smoking patterns could lead to potential implications for prevention and treatment (33). In this regard, a comprehensive assessment will help to identify possible inter-subject variables that may be associated with a pattern of sustained tobacco abuse. In response to these limitations, we conducted a naturalistic and cross-sectional study to identify key factors that promote nicotine use. Particularly, this study was designed to determine the effects of several executive control functions. Assuming the three components of the dominant executive functioning model (18), we assessed inhibitory processes, updating and shifting on clinical aspects of smoking. In agreement with existing studies, we hypothesized that inhibition and updating components would be associated with years of addiction. Furthermore, we expected to find that long-term smokers should report more signs of executive dysfunction.

## Materials and Methods

The sample consisted of 174 smokers (59% females), whose age ranged between 27 and 69 years old (M = 47.44; SD = 8.48) and they were recruited across 3 years. The average sample score in Fagerström Test for Nicotine Dependence (FTND) was 4.49 (SD=2.32) and they smoked an average of 17.9 (SD=8.94) cigarettes per day. Participants were eligible if they were current smokers and the sample selection criteria were: (1) aged above 18 years old (2) were employed by the University of Granada (Spain). The exclusion criteria were: (1) any illness or mental disorders suggesting possible difficulty in completing the different tasks and (2) current psychotropic medication for psychiatric symptoms, concurrent dependence on other substances (cocaine, heroin, alcohol, etc.). All the participants signed a written consent form. The study protocol was approved by the Bioethics Committee of the University of Granada (Spain) and adhered to the tenets of the Declaration of Helsinki.

### Experimental Design

We conducted a naturalistic and cross-sectional study during the course of a smoking cessation treatment intervention. Smokers were invited to participate in a study assessing personality and cognition in relation to smoking behavior. Participants were recruited as they engaged in an occupational health service that provides smoking cessation treatment including pharmacological (varenicline) and behavioral change components. This treatment consisted of three phases: (1) psychoeducation phase to reduce smoking (planning of activities and establishment of objectives), (2) prescription and controlled administration of the drug varenicline a partial agonist and antagonist drug in the presence of nicotine neuronal receptors for nicotine acetylcholine α4β2 and (3) training of relapse prevention strategies.

The program begins with an initial session where a semi-structured interview for smokers is conducted as well as a neuropsychological assessment of all smokers. All measures of this study were collected at baseline. Tests were administered always in a fixed order designed to alternate between easy/difficult tasks. This initial evaluation was conducted in a single session, considering the appropriate breaks to avoid the effect of fatigue. A unique code was assigned to the participants in order to carry out an individual follow-up while safeguarding anonymity.

### Measurements

All neuropsychological evaluations were conducted by trained psychologists. Smokers were evaluated at baseline with these assessments:

#### Semi-structured Interview for Smokers (34)

This survey provides information about socio-demographic data, smoking duration, level of dependence and brand of cigarettes. The primary outcome variable was assessed. Years of smoking addiction were defined as the number of years from the initiation of nicotine use to the initiation of smoking cessation treatment. Another important parameter to consider when evaluating higher prevalence of smoking is the number of cigarettes consumed per smoking day (CPD). CPD was used as a covariate in analyses.

#### Fagerström Test for Nicotine Dependence (20)

This test was designed to provide an ordinal measure of nicotine dependence related to cigarette smoking. It contains six items that assess the quantity of cigarette consumption, the compulsion to use and dependence. The FTND produces a score from 0 to 10, with higher scores indicating more severe nicotine addiction. The FTND allows classification of nicotine dependence severity into different levels: low (0 to 2 points), moderate (3 to 7 points), and high (>7 points).

#### Letter-Number Sequencing Task (28)

This task is an attentional subtest of the Wechsler Adult Intelligence Scale. The participant must sequence a random order of numbers and letter. They must say the numbers in ascending order and then the letters in alphabetical order. The dependent variable to be considered in this test would be the total number of hits.

#### Visual Search and Attention Test (35)

This test consists of four visual cancellation tasks that require the respondent to cross out letters and symbols that are identical to a target. It is an attention task that require visual search based on either a single-feature search or a dual-feature search. The main dependent variable used in this test was number of correctly identified targets.

#### Go/No Go Task (36)

This task assesses the ability to inhibit a simple motor response. It consisted of 60 trials. In the first 30 trials (pre-switch), participants were asked to press a key as quickly as they could whenever the go stimulus (a letter) was presented, and to withhold the response when the no-go stimulus (a different letter) was presented. In the second 30 trials of the task (post-switch), participants were asked to respond to the previously no-go stimulus and not to respond to the previously go stimulus. The proportion of go vs. no-go trials on both phases (pre- and post-switch) was 7/3. The inter-stimulus interval (ISI) was set at 100 ms, and each stimulus was presented during 1,000 ms. Auditory feedback was provided after each response to indicate whether that response had been right or wrong. Responses were coded as hits (responding in presence the go trial), false alarms (responding in presence of the no-go trial), misses (not responding in presence of the go trial), and correct rejections (not responding in presence of the no-go trial). The main variable from this task was the false alarm rate, computed as the ratio between the number of false alarms and the total number of no-go trials.

#### Delay Discounting Task (37)

This task is a monetary-choice questionnaire asking for individual preferences between smaller, immediate rewards and larger, delayed rewards varying on their value and time to be delivered. The questionnaire is composed of a fixed set of 27 choices. We calculated the area under the curve (AUC) (38). The AUC was calculated for the range of reward magnitudes included in the questionnaire (small –Euro 25 to 35; medium –Euro 50 to 60; and large –Euro 75 to 85), according to the formula (x2–x1) [(y1–y2)/2], where x1 and x2 are successive delays, and y 1 and y 2 are the subjective values associated with these delays.

#### Iowa Gambling Task (39)

The task is a computerized measure of decision-making abilities. The participants attempt to win as much play money as possible by selecting cards from four decks (A, B, C, and D). Each time a participant selects a card, a specified amount of play money is awarded. However, interspersed among these rewards, there are probabilistic punishments (monetary losses). Two of the decks of cards (A and B) produce high immediate gains; however, in the long run, they will take more money than they give, and are thus considered disadvantageous. The other two decks (C and D) are considered advantageous, as they result in small, immediate gains, but will yield more money than they take in the long run. The decisive dependent measure for this task was the difference in the number of cards selected from the advantageous vs. disadvantageous decks across five block of 20 trials.

#### Five Digit Test (40)

This test is a numerical Stroop task divided into four components. The first component demands participants to name numbers from 1 to 5 as fast as they can. On the second component, participants must describe quantities from 1 to 5. The third component involves a selective attention trial, they must not read the numbers but rather tell how many numbers are present in each stimulus. Finally, on the fourth component participants must read the stimulus numbers. We recorded the total time for each component. The main dependent variable used in this test was the time required to complete each task.

### Statistical Analysis

We presented sociodemographic data as percentages and frequencies. In addition, we tested the association of years of smoking with other neuropsychological tests using Spearman rank-order correlations. Firstly, we performed a linear regression analyses including the following predictors: Letter-Number Sequencing Task total score, Visual Search and Attention Test total stimulus score, Go/No Go false alarms, Delay Discounting area under the curve, Iowa Gambling Task net score and Five Digit Test score. The dependent variable was the years of smoking addiction. Secondly, we performed a multivariate analysis of variance (MANCOVA) to determine whether there are any differences between performance in cognitive tasks and relevant aspect of smoking. Although there is not a consensus on how to best define smoking (31), different variables collected by smoking history were considered to provide a broader assessment of smoking (32). In consequence, smokers were coded as either “light smoking” (0–24 years) or “heavy smoking” (≥ 25 years) on the results of smoking behavior self-reports. Nicotine dependence level as measured by FTND were coded as high level of dependence (>7 points) and moderate level of dependence (3 to 7 points) (20). Finally, according to the number of cigarettes per day, it has been proposed that smokers can be divided into three levels (CPD: 5–14, CPD: 15–24, ≥25 CPD) (41).

All statistical tests were performed using the software package SPSS version 25.0 (42). We performed an analysis on all the participants simultaneously. For all analyses, *p* = 0.05 was adopted as the significance criterion.

## Results

The means and standard deviations of clinical aspects of smoking and neuropsychological measures are provided in Table 1. The mean sample score on the Fagerström test for nicotine dependence (FNDT) was moderate (M = 4.49, SD = 2.32). The sample had an average of 17.9 cigarettes per day (SD = 8.94) with an average nicotine level per cigarette of 0.99 mg (SD=.13). Besides, participants showed a low level of previous attempts to quit smoking (M = 1.27, SD = 1.35) during their years of addiction. Men smokers reported a significantly higher level of both nicotine dependence on the FTND overall (M = 5.05, SD = 2.51) and cigarettes per day (M = 20.96, SD = 10.02) that did woman smokers (M = 4.19, SD = 2.05; M = 16.20, SD = 7.92).

**Table 1:** Baseline demographic and smoking characteristics of the participants (*n* = 174).

	**Variables**	**Mean**	**SD**	**Range**
	Age	47.3	8.31	27–69
	Years of schooling	17.13	5.40	8–25
**Smoking characteristics**				
	Fagerström Test for Nicotine Dependence	4.49	2.32	0–10
	Years of smoking addiction	28.43	9.84	4–57
	Number of daily cigarettes	17.9	8.94	2–60
	Level of nicotine	0.99	0.13	0.60–1.8
	Attempts to quit smoking	1.27	1.35	0–12
**Neurocognitive variables**				
	Letter-Number Sequencing Task	8.64	2.98	0–15
	Visual Search and Attention Test	228.13	58.26	18–383
	Go/No Go Task	5.67	6.60	0–50
	Delay Discounting Task	0.57	0.22	0–1
	Iowa Gambling Task	−0.882	26.11	−86–78
	Five Digit Test	14.49	8.76	−33–43

*Descriptive scores for socio-demographic variables including age, years of education, smoking characteristic evaluated by the Semi-structured Interview for smokers (34), level of nicotine (mg per cigarette) and neurocognitive variables*.

Table 2 shows the pattern of correlations between Years of smoking addiction and neuropsychological measures. Moderate correlations were found between Years of smoking and measures of working memory (VSAT), inhibitory control (GNG) and cognitive flexibility (IGT), ranging from −0.119 to −0.425. The strongest association was with the working memory score (VSAT). We also found a differential pattern of association of the variable outcome with Letter-Number Sequencing Task, Delay Discounting Task and Five Digit test (ranging from −0.011 to 0.078).

**Table 2:** Pearson correlation analysis between cognitive variables and Years of smoking (*n* = 174).

**Variables**	**Years**	**WAIS**	**VSAT**	**GNG**	**DDT**	**IGT**	**FDT**
Years	1						
WAIS	−0.011	1					
VSAT	−0.425^*^	0.177^*^	1				
GNG	0.122^*^	−0.029	−0.123^*^	1			
DDT	0.078	−0.017	−0.045	−0.039	1		
IGT	−0.119^*^	0.020	0.039	−0.034	0.072	1	
FDT	−0.074	−0.249^*^	−0.152^*^	0.165^*^	−0.064	0.079	1

* *p < 0.05. Years, Years of smoking addiction; WAIS, Letter-Number Sequencing Task; VSAT, Visual Search and Attention Test; GNG; Go/No Go Task; DDT, Delay Discounting Task; IGT, Iowa Gambling Task; FDT, Five Digit Test*.

After bivariate correlation analysis, multiple regression analyses were used to identify predictors of years of smoking addiction in current smokers. The model including neurocognitive scores showed satisfactory fit explaining 22.2% of variance, *F*_(6, 167)_ = 7.893; *p* < 0.000. Inspection of parameter estimates showed that the dimension of working memory (VSAT) was significantly and inversely associated with Years of smoking (β = −0.434, *p* < 0.000). However, none of the others neurocognitive tasks were significantly associated with the primary outcome variable (see Table 3).

**Table 3:** Multiple linear regression analysis (*n* = 174).

**Variable**	**β**	***t***	***p***	**95% IC**
WAIS	0.038	0.59	0.597	−0.348/0.603
VSAT	−0.434	−6.16	0.000	−0.095/−0.049
GNG	−0.091	1.30	0.193	−0.067–0.330
DDT	0.061	0.88	0.378	−3.171–8.318
IGT	−0.094	−1.36	0.174	−0.085–0.016
FDT	−0.134	−1.84	0.066	−0.300–0.010

* *p < 0.05. WAIS, Letter-Number Sequencing Task; VSAT, Visual Search and Attention Test; GNG; Go/No Go Task; DDT, Delay Discounting Task; IGT, Iowa Gambling Task; FDT, Five Digit Test*.

Years of addiction (0–24 years; ≥ 25 years) x FTND level (moderate level of dependence or high level of dependence) x cigarettes per day (CPD: 5–14, CPD: 15–24, ≥ 25 CPD) multivariate analysis of covariance (MANCOVA) was conducted on the WAIS subscale, the VSAT test, the GNG task, the DDT task, the IGT and the FDT with age as the covariate. According to Pillai's Trace multivariate effects were highly significant for the FTND *F*_(7, 156)_ = 2.107, *p* < 0.041, partial η2 = 0.086. Univariate effects on VSAT was significant *F*_(11, 156)_ = 8.455, *p* < 0.000, and also on WAIS, *F*_(11, 156)_ = 1.877, *p* < 0.046 (Table 4). To further clarify the findings, Tukey *post-hoc* test (*p* < 0.05) indicated that long-term smokers (≥25 years) scored significantly lower (indicated worse functioning) on VSAT task. In addition, both smokers with higher levels of dependence on FNDT (>7 points) and smokers with higher rates of cigarettes per day (≥25 CPD) score worse on WAIS task.

**Table 4:** Tests of between-subjects effects.

	**Variable**	**Type III Sum of Squares**	**df**	**Mean Square**	***F***	**Sig**.	**Partial Eta Squared**
Corrected Model	WAIS	165.709	11	15.064	1.877	0.046	0.113
	VSAT	22,788.21	11	20253.4	8.455	0.000	0.365
	GNG	712.205	11	64.746	1.434	0.162	0.089
	DDT	0.455	11	0.041	0.753	0.686	0.049
	IGT	8,624.334	11	785.03	1.116	0.352	0.070
	FDT	774.520	11	70.411	0.851	0.590	0.055

* *p < 0.05. WAIS, Letter-Number Sequencing Task; VSAT, Visual Search and Attention Test; GNG; Go/No Go Task; DDT, Delay Discounting Task; IGT, Iowa Gambling Task; FDT, Five Digit Test*.

## Discussion

Through this behavioral research, we intend to explore possible individual differences that may be linked to an executive pattern attributable to sustained smoking abuse. The aim of this study was to identify predictors of years of smoking addiction in a sample of current smokers. Specifically, we investigated the influence of neurocognitive variables such as updating, inhibitory control and cognitive flexibility ability. Results from the analysis revealed that the regression model including neurocognitive scores showed satisfactory fit explaining 22.2% of variance. However, an inspection of parameter estimates showed that the dimension of working memory (VSAT)was significantly and inversely associated with Years of smoking addiction. In consequence, the main finding of this study was that a poorer performance in working memory predicts the primary outcome variable. These results are in line with some previous scientific studies which examined the relationship between smoking and cognitive performance in working memory. Sutherland et al. (43) analyzed the effect of nicotine on EF performance through a working memory task based on the continuous-counting paradigm that required maintenance and simultaneous shifting in a sample of smokers and non-smokers. The findings suggest that smokers showed increased sustained activation in the medial and lateral prefrontal cortex during task execution. Smokers were also less effective in mobilizing the cognitive resources required to perform tasks that require recruitment and control of working memory. Thus, the increase in frontal activity recorded in smokers represents the need to increase cognitive resources to guide behavior during task execution (43). Recent data suggest that smokers performed worse on some cognitive tasks (arithmetic and digits subtests) (28) than nonsmokers. In addition, working memory (scores on digit subtest) was related to the age of starting smoking (27). In contrast, the lack of findings among others neuropsychological measures could be attributed to the fact that executive components such as inhibition and change are involved in other aspect of nicotine addiction such as relapse or initiation. For example, López-Torrecillas et al. (44) analyzed the predictive capacity of different temperamental measures [Barratt Impulsiveness Scale (44)], temperamental dimensions [Temperament and Character Inventory Revised; (45)], and neurocognitive tasks: Go/NoGo task (36), a delayed discount task (37) and decision making [Iowa Gambling Task; (39)] to predict smoking relapse. The results showed that poorer performance on the Gambling Task predicted greater relapse (44). Besides, a recent study (46) examined the predictive ability of delayed discounting to predict relapses after six months of tobacco cessation. The logistic regression model reported that longer discount delay, younger age, more previous quit attempts and higher dependence (FTND) were associated with higher risk of relapse at the 6-month follow-up.

On the other hand, multivariate analyses of covariance (MANCOVA) revealed highly significant differences between heavy smokers and light smokers on both VSAT and WAIS tasks after controlling for age via covariate analysis. When age scores were added as covariate in the MANCOVA, the observed differences between light and heavy smokers seemed potentially attributable to the higher levels of nicotine dependence in the heavy smokers. In particular, nicotine dependence level as measured by FTND significantly interacted with updating measures. Sensitivity analyses further showed that long-term smokers (≥25 years) scored significantly lower on the VSAT task. Besides, groups with both highest levels of dependence on FNDT (>7 points) and highest rates of cigarettes per day (≥25 CPD) showed worse scores on WAIS task. Group differences in working memory may reflect the injurious effects of smoking. Also, these results indicate that updating measures could be useful to prospectively predict years of addiction, such that they could be utilized to identify potential heavy smokers. Consequently, based on human neuroimaging evidence, the insula is critically involved in maintaining cigarette smoking (47). Recently, Li et al. (48) investigated the differences in insular morphometry between smokers and non-smokers. The authors explained that the insula had a trend to be negatively correlated (not significant) with pack-years. However, they found decreased cortical thickness of insula in smokers compared to non-smokers (48).

The analyses presented in this article emphasize the need to include a comprehensive smoking assessment, which contains variables of smoking behavior, to examine the effects of nicotine consumption on cognition. As mentioned before, the diverse behavioral effects of nicotine on different aspects of cognition suggest that there is considerable heterogeneity in the underlying causes of smoking (4). Taken together, the results highlight key factors that promote nicotine use. Smoking behavior may reflect a decrease in working memory and attention span that requires a visual search. Following Kübler et al. (49), we hypothesize that smokers with a long history of tobacco addiction show poor performance in working memory and attention tasks then it predisposes them to ruminative and craving thoughts, conventionally linked to addiction (49, 50).

This study has some important limitations that should be considered for an appropriate interpretation of its findings. First of all, participants were all employees of the same institution and there may be a concern because the sample is not representative of clinical populations but sufficiently diverse to be representative of the general population. Secondly, the cross-sectional nature of this study makes it difficult to obtain the inferred causal relationship between the variables. However, to our knowledge, this is the first study to show that a cognitive measure (attention task that requires visual search) is significantly associated with years of smoking. Despite these limitations, these findings have important public health implications and suggest that interventions designed for smoking cessation should focus not only on reducing relapses but also on improving executive functions such as working memory and attentional bias to smoking cues. In addition, these findings suggest that cigarette packaging warning (51) may experience a loss of effectiveness in long-term smokers as well as high levels of dependence. In this sense, behavioral approaches that recover cognitive functions help to improve theoretical support (33). Future studies in this area will have critical value toward reducing deficits in executive control functions, resulting in public health benefits.

## Data Availability Statement

The raw data supporting the conclusions of this article will be made available by the authors, without undue reservation.

## Ethics Statement

The studies involving human participants were reviewed and approved by Bioethics Committee of the University of Granada (Spain). The patients/participants provided their written informed consent to participate in this study.

## Author Contributions

RMR and FLT: conceived, designed, and performed the experiments and wrote the paper. RMR, FLT, and IMT: analyzed the data. RMR: guarantor for this paper. All authors: read and approved the final manuscript.

## Conflict of Interest

The authors declare that the research was conducted in the absence of any commercial or financial relationships that could be construed as a potential conflict of interest.
